# Monitoring the Impact of Two Pedagogical Models on Physical Load in an Alternative School Sport Using Inertial Devices

**DOI:** 10.3390/s25185929

**Published:** 2025-09-22

**Authors:** Olga Calle, Antonio Antúnez, Sergio González-Espinosa, Sergio José Ibáñez, Sebastián Feu

**Affiliations:** 1 Training Optimization and Sport Performance Research Group (GOERD), Sport Science Faculty, University of Extremadura, 10005 Caceres, Spain; olcallem@unex.es (O.C.); antunez@unex.es (A.A.); sibanez@unex.es (S.J.I.); sfeu@unex.es (S.F.); 2 Faculty of Sport Sciences, University of Extremadura, 10003 Caceres, Spain; 3 NÌKE: Research Group in Education, Health and Sports Performance, Didactics of Physical Education and Health, International University of La Rioja (UNIR), 26006 Logroño, Spain; 4 Faculty of Education and Psychology, University of Extremadura, 06006 Badajoz, Spain

**Keywords:** physical–physiological demand, pedagogical models, physical education, alternative invasion sport, inertial device

## Abstract

(1) Background: Physical Education sessions subject students to various physical and physiological demands that teachers must understand to design interventions aimed at improving health and fitness. This study aimed to quantify and compare external and internal load before and after implementing two intervention programs: one based on the Game-Centered Model and another Hybrid Model that combines the Game-Centered Model with the Sport Education Model. (2) Methods: A total of 47 first-year secondary school students participated, divided into two naturally formed groups. Pre- and post-intervention assessments involved 4 vs. 4 matches monitored using WIMU Pro™ inertial measurement units and heart rate monitors to collect kinematic, neuromuscular, and physiological data. The combined use of inertial sensors and heart rate monitors enabled the objective quantification of students’ physical demands. (3) Results: No significant improvements were observed between pre- and post-tests, possibly due to the short duration of the interventions. However, the Hybrid Model generated higher levels of external load, both kinematic and neuromuscular, in the post-test. (4) Conclusions: The Hybrid Model appears more effective in increasing students’ physical engagement. Inertial sensors represent a valid and practical tool for monitoring and adjusting instructional strategies in school-based Physical Education.

## 1. Introduction

During childhood, Physical Education (PE) constitutes a foundational element of development across the school years, marking the initial structured exposure to physical activity and sport. Consequently, PE is widely recognized as a critical platform for fostering lifelong engagement in physical exercise among students [1,2]. Consistent participation in physical activity is well established as a determinant of both physical and psychological health outcomes [3]. Contemporary PE curricula increasingly prioritize student health, aligning with public health objectives. Given the prominent role of sport in modern society, its integration into PE programs is considered particularly effective, as it captures student interest, promotes sustained participation in physical activity, and facilitates the adoption of active lifestyles [4].

School-aged children are recommended to engage in at least 60 min of moderate to vigorous physical activity daily to ensure optimal cardiovascular health [5]. However, the time dedicated to vigorous activity during PE classes is limited [6], accounting for only 10% of total session time, which translates to approximately five minutes [7]. Understanding the physical demands elicited by PE practices is essential for enhancing the health-related aspects of student development.

Alternative games and sports constitute a social movement encompassing a space of non-conventional physical and sport-related practices, which are in constant evolution through the creation of new games and sports, as well as the hybridization, modification, and revival of traditional games and sports. In this context, the playful component is prioritized over competitive sports performance [8]. Within the Spanish educational context, there is a growing trend toward implementing alternative sports due to their educational potential [8], as they can be adapted to align with intended pedagogical objectives [9,10]. These sports not only promote comprehensive student development [11] but also facilitate equitable access, participation, and sporting practice, due to their undefined, mixed, and flexible characteristics. These modalities are well-suited to schoolchildren’s motor and physical capacities and demonstrate high adaptability to the various spaces and materials available in educational centers [8,9,12]. The implementation of alternative sports through appropriate methodology provides meaningful learning in both declarative and procedural knowledge [13]. However, little is known about how alternative sports practice influences physical fitness levels and the extent of physical load imposed on students.

Sport pedagogy is grounded in pedagogical models that provide the scaffolding structures necessary for appropriately integrating teaching techniques, styles, and strategies. These models are understood as long-term approaches essential for achieving pedagogical objectives [14,15,16]. Therefore, teachers must select and apply appropriate pedagogical models to facilitate the acquisition of targeted competencies [17]. The methodology employed largely determines students’ physical conditioning [18], as it results from the configuration of pedagogical, organizational, and task load variables. Active models, such as comprehensive sport teaching models, have been demonstrated to yield superior outcomes in sport learning compared to traditional models (direct instruction) [19].

The Game-Centered Model (GCM) promotes student involvement and participation, elements that lead to enhanced game understanding, tactical learning, and decision-making [20,21]. The Game-Centered Model (GCM) falls within the framework of the tactical games approach to sports education. It aims to develop sport-specific skills and technical–tactical knowledge through a game-contextualized approach that promotes active student engagement. The teacher implements modified and global sports games combined with interrogative feedback to enhance students’ tactical intelligence and decision-making skills [21]. The methodological approach adopted by teachers impacts students’ physical fitness, as it is associated with different load response patterns [22]. Comprehensive Sport Teaching Models, including the GCM, facilitate the development of motor skills and physical fitness levels, proving effective for learning invasion sports [23].

The Sport Education Model (SEM) is grounded in competency-based teaching, aiming to promote authentic sport experiences through the performance of various roles and responsibilities. It is implemented across several phases: affiliation, preseason, regular y final competition, performance record keeping, and culminating festivity or event [24]. This model allows for multiple pedagogical perspectives when designing learning tasks, ranging from traditional (reproductive) to active (inquiry-based) approaches. The Sport Education Model (SEM) has demonstrated benefits in psychological and social variables, technical–tactical learning, and students’ physical capabilities [24,25].

The hybridization of the Game-Centered Model and the Sport Education Model (HM) integrates tasks based on the Game-Centered Model (GCM), specifically the use of modified and global sports games with guided inquiry and feedback, alongside the implementation of key phases, including affiliation, preseason, regular and final competition, performance record keeping, and culminating events, as well as sport-related roles [13]. The application of this hybrid model has demonstrated improvements in game understanding, student motivation, and skill development [26,27,28]. In the context of analyzing physical activity intensity during Physical Education classes, the Game-Centered Model has been shown to elicit significantly higher levels of physical engagement compared to the Hybrid Model (HM) [29]. However, current evidence is insufficient to determine how this methodology affects physical activity levels within school educational settings.

The measurement of external load (eTL) and internal load (iTL) can be conducted using various instruments that enable understanding of the physical and physiological demands sustained during tasks. External load is defined as the mechanical and locomotor stress generated during physical activity (imposed stimulus), comprising neuromuscular and kinematic loads [30]. Objective quantification can be achieved through inertial devices that integrate sensors such as accelerometers, gyroscopes, barometers, and GPS [31,32]. However, due to their high cost, these devices are often inaccessible to schools and are more commonly used in sports performance contexts. In contrast, subjective evaluation methods such as the Integral System for Training Task Analysis (SIATE) have been validated in studies for measuring subjective loads [33].

Internal load (iTL) assessment is conducted through heart rate monitoring or subjective perception scales [34], enabling estimation of physical effort intensity [35]. In this context, iTL represents the physiological response and stress experienced because of physical stimulus, primarily assessed through heart rate [36]. Objective measurement is performed using heart rate monitors, while subjective evaluation typically involves the Rating of Perceived Exertion (RPE) scale [37]. The integration of these technological tools in educational settings enhances teaching–learning process quality by enabling task adjustment to actual physical demands, thereby promoting cardiovascular health and comprehensive physical development [6]. Additionally, it facilitates cross-validation of subjective methods within school environments [38].

In the context of sports monitoring, inertial devices are not strictly limited to technologies that measure inertial physical quantities such as accelerations or rotations but encompass integrated sensor systems that enable the quantification of both external load and athletes’ internal and tactical variables. Specifically, an Inertial Measurement Unit (IMU) is defined as a device that combines multiple motion-tracking technologies, typically including accelerometers, gyroscopes, and, occasionally, magnetometers, with the purpose of capturing dynamic movement parameters [39]. This multidimensional approach allows technologies such as GPS, LPS, or UWB systems to be integrated into monitoring platforms which, despite also employing positioning methods, are identified as inertial devices in sports applications due to their combined function of collecting data on external, internal, and tactical loads [40].

In recent years, the use of sensors has transformed the assessment of physical demands in both educational and sports contexts. Inertial measurement devices, such as the WIMU Pro™ system (Real Track Systems, Almería, Spain), which integrates accelerometry, gyroscope, and magnetometry sensors, enable highly accurate quantification of variables related to external load (eTL) and locomotor-cinematic internal load (iTL) experienced by students during Physical Education sessions [6,38]. Regarding external load, sensors collect information based on mathematical formulas, processing this data, and converting it into manageable variables for sports professionals. Additionally, internal load is obtained via a Bluetooth connection using heart rate bands. This technology not only enables movement pattern analysis but also facilitates individualized instruction and task design optimization according to actual physiological demands. Furthermore, sensor integration in school settings contributes to validating subjective effort evaluation tools, expanding possibilities for objective measurement without interfering with natural activity flow [18,33]. Their implementation is particularly useful in studying alternative sports, where stimulus variability and novel gameplay dynamics require sensitive, reliable, and adaptable monitoring methods for various spatial and material configurations.

“Rosquilla” is an alternative invasion sport created within the school context, played on a rectangular field that includes two circular scoring zones. It utilizes a PVC hoop as the primary implement and is characterized by promoting equal participation, cooperation, coeducation, and adaptability to different school contexts, requiring only basic technical skills and accessible materials [41]. Most studies analyze the effects of teaching methodologies in relation to game understanding (declarative and procedural knowledge) [13], as well as psychological and pedagogical variables [42]. However, few studies explore the physical and physiological demands generated in school settings following the implementation of different methodologies for sports learning. This research gap represents not only a theoretical limitation but also a practical constraint for teachers seeking to implement inclusive and motivating content in their educational programming. Most existing studies are situated within sports performance contexts [43]. In the educational field, some studies have assessed internal and external load in basketball [6,18] and football [38] learning according to different methodologies. However, no studies have analyzed the physical–physiological demands in alternative invasion sports based on the pedagogical model employed.

To compare the effects of implementing different methodologies on students, it is necessary to examine differences in physical and physiological demands experienced by students under various methodological approaches. This need becomes even more relevant when extended to alternative sports, which are frequently utilized by teachers in Spain yet lack sufficient evidence regarding their impact on student learning. This study analyzes and compares the physical and physiological demands of two different intervention programs.

The objectives of this study were (i) to quantify the physical and physiological demands (eTL and iTL) experienced by students while playing the alternative sport “Rosquilla” before (pre-test) and after (post-test) implementing two pedagogical models—the Game-Centered Model (GCM) and the Hybrid Model (HM); (ii) to compare load quantification according to the pedagogical models; and (iii) to compare quantification based on evaluation tests. It is hypothesized that the Hybrid Model (HM) will generate higher physical loads (eTL and iTL) than the GCM due to its more intensive and participatory methodological structure. The results of this research may guide Physical Education teachers in selecting more effective methodologies to optimize students’ physical load and promote active habits from an early age.

## 2. Materials and Methods

### 2.1. Research Design

A quasi-experimental design was employed using two groups with repeated measures (pre-test and post-test) [44] to analyze the physical and physiological demands of students before and after the implementation of two differentiated instructional approaches: the Game-Centered Model (GCM) and the Hybrid Model (HM), during the learning of an alternative invasion sport known as the “Rosquilla”. Two intervention programs were developed and validated [45]. It is worth noting that this was the students’ first experience both with the alternative sport the “Rosquilla” and with the pedagogical models—specifically the GCM and the hybridization of the GCM and Sport Education Model (SEM). The intervention lasted for two months.

### 2.2. Participants

Participants in the study were selected through non-probability convenience sampling [44], due to feasibility and accessibility considerations for the researchers. The sample consisted of two intact classes comprising a total of 47 first-year secondary school students (26 girls and 21 boys), aged between 12 and 13 years (*M* = 12.41; *SD* = 0.59), from a public educational institution located in the southwest of Spain. These natural groups were divided as follows: Class A, composed of 26 students (10 boys and 16 girls), and Class B, composed of 21 students (11 boys and 10 girls). Two incidental and natural groups from the Spanish educational system were selected. In this Spanish educational system, groups are organized by the educational administration based on educational and equity criteria, resulting in a mixed and heterogeneous configuration. Therefore, it was decided to respect the criteria established by the educational administration regarding group composition to ensure the ecological validity of the study. Class A was randomly assigned to the Hybrid Model (HM) methodology, while Class B was assigned to the Game-Centered Model (GCM). Consequently, the results may be influenced by the natural structure of the research groups.

Inclusion criteria for participation in the study were (1) obtaining informed consent from parents or legal guardians; (2) attending at least 80% of the sessions; and (3) completion of both pre-test and post-test assessments. The initial sample included 56 students; however, 9 participants were excluded for not meeting the inclusion criteria, resulting in a final sample of 47 students.

The sample used in this study exhibits a high degree of ecological validity, as it comprises intact, non-manipulated natural groups. This ensures that the results accurately reflect the real conditions of the educational context under investigation. However, this same characteristic limits external validity, as the results of the inferences will primarily be applicable to contexts with similar characteristics (i.e., the same educational level, class organization, and practice of the sport “Rosquilla”). Nevertheless, the sample size was calculated a priori using G*Power version 3.1.9.7 for the Mann–Whitney U test, setting the effect size at d = 1.2 (very large), a significance level of α = 0.05, and an observed power (1 − β) = 0.95. The software (G*Power version 3.1.9.7) indicated a minimum requirement of N = 34 observations (17 per group). Ultimately, the actual sample reached 47 cases (26 in Group A and 21 in Group B), exceeding the initial estimate and thereby ensuring the statistical power required for the planned analyses.

### 2.3. Variables

The independent variable corresponded to the pedagogical models: the Game-Centered Model (GCM) and the Hybrid Model (HM), which integrates elements of both the Game-Centered Model and the Sport Education Model (SEM). The dependent variables were the students’ physical and physiological demands. Specifically, a total of 21 objective dependent variables were recorded, grouped into external training load (eTL) and internal training load (iTL) indicators. Within the eTL category, a distinction was made between kinematic and neuromuscular variables.

**Figure 1:**
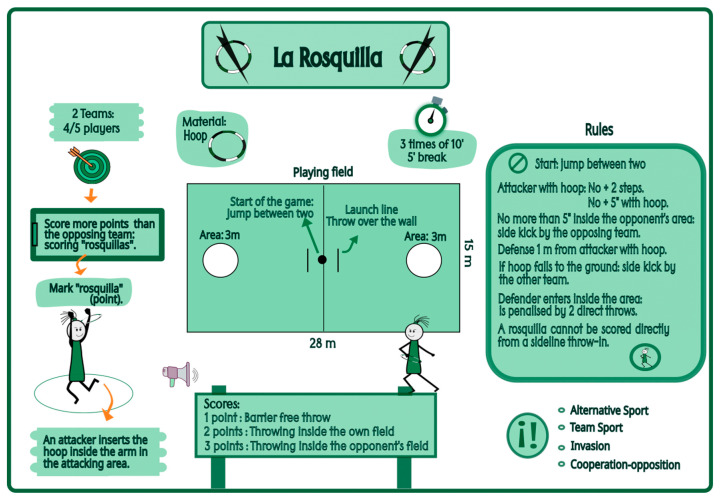
The characteristics of the alternative sport the Rosquilla. Note: Adapted from Calle et al. [45].

Regarding the independent variable, two differentiated instructional methodologies were considered: the Hybrid Model (HM) for Group A and the Game-Centered Model (GCM) for Group B. The intervention programs were similar in terms of content, learning objectives, phases of play, and types of learning tasks, but differed according to the specific pedagogical features of each model. In the HM, students engaged in the distinct phases of the Sport Education Model (SEM) and were granted autonomy to select and manage the development of tasks through assigned roles during the student-directed phase (sessions 7–12). Both intervention programs were validated by experts in invasion sports and in the application of the respective pedagogical models. They obtained excellent values for content validity (*Aiken’s V* ≥ 0.73) and internal consistency (*Cronbach’s α* = 0.99) through evaluation by a panel of nine expert judges [45]. The results demonstrated that both programs are valid and reliable for teaching the “Rosquilla” in PE settings. The characteristics of the alternative sport the “Rosquilla”, created by Professor Manuel Rodríguez-Barriga [41], are included in Figure 1.

Both intervention programs were delivered by the lead researchers, who possessed formal training and experience in alternative invasion sports and pedagogical models. Additionally, the researchers received support from a panel of experts in pedagogical models, all holding doctoral degrees, who provided guidance and consultation throughout the intervention period.

The dependent variables analyzed in the study were grouped into external training load (eTL) and internal training load (iTL) indicators. Within the eTL, two categories were distinguished: kinematic and neuromuscular variables. The kinematic eTL variables included: distance covered in meters (dis(m)); explosive distance (dis-exp(m)); meters per minute (m/min); number of accelerations (Nacc); accelerations per minute (acc/min); number of decelerations (Ndec); decelerations per minute (dec/min); maximum speed (MAX Speed (Km/h)); average speed (AVG Speed (Km/h)); number of sprints (Nsprints); number of high-speed runs exceeding 18 km/h (HSR); number of steps (Nsteps); steps per minute (steps/min); number of jumps (Njumps); and jumps per minute (jumps/min). The neuromuscular eTL variables comprised the number of impacts sustained (Nimpacts), Player Load (PL; arbitrary units (au)), and Player Load per minute (PL/min). Regarding internal training load (iTL), the variables considered were maximum heart rate (HRmax; beats per minute (bpm)), average heart rate (HRavg; beats per minute (bpm)), and relative percentage of maximum heart rate (%HRrel). For each variable, both the absolute value (total number of actions) and the relative value (actions per minute) were recorded, to provide a complementary perspective that combines total volume with the relative frequency of execution based on actual participation time.

### 2.4. Instruments

The eTL and iTL variables were recorded before and after the implementation of the pedagogical programs using WIMU Pro™ inertial devices (Real Track Systems, Almería, Spain) [32] in combination with GARMIN™ heart rate monitors (Garmin Ltd., Olathe, KS, USA). The WIMU Pro™ system is a validated multicomponent inertial measurement unit (IMU) that integrates a triaxial accelerometer, gyroscope, magnetometer, barometer, GPS, and UWB antennas, enabling precise quantification of locomotor and neuromuscular behavior in dynamic environments, including indoor settings without satellite coverage [6,31]. This compact and lightweight device is positioned on the upper back, specifically, in the interscapular line, within an ergonomic vest, ensuring it does not interfere with the students’ motor performance [32].

**Figure 2:**
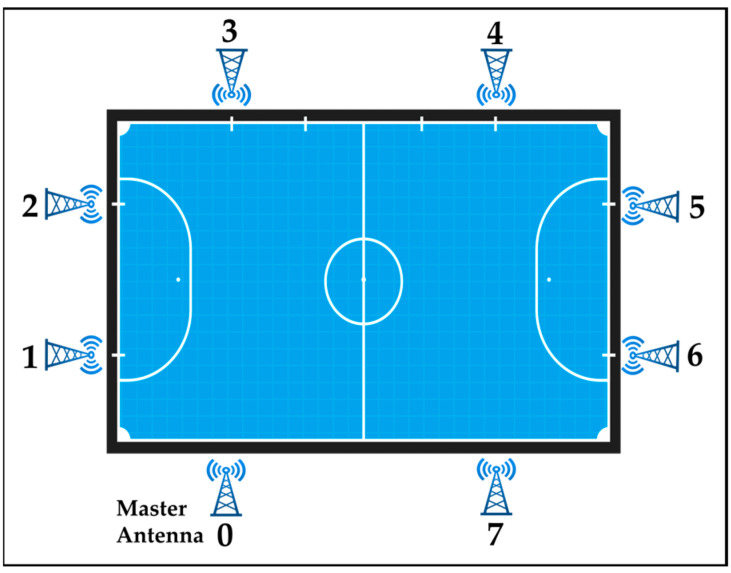
Placement of UWB antennas on the playing field, during the pre-test and post-test assessment sessions.

The sensors were programmed to record data at a sampling frequency of 100 Hz for accelerometry and gyroscope, capturing broad, gross motor movements involving large muscle groups. For data recording, Ultra-Wideband (UWB) technology was employed by placing eight antennas around the sports field (Figure 2), as it provides greater accuracy and reliability compared to data collected using the Global Positioning System (GPS) [46]. Consequently, an ultra-wideband (UWB) system was employed for positional tracking. UWB radiofrequency-based technology offers reliable positioning data in diverse environments, both indoor and outdoor, with efficacy in indoor settings where GPS functionality is compromised, and other radiofrequency systems encounter accuracy limitations. The precision of UWB tracking systems is regarded as sufficient for applications in sports analysis. Moreover, position estimations provided by UWB technology are highly accurate and deemed appropriate for detailed sports performance evaluations [47]. Additionally, the ANT+ system was used to synchronize the real-time positioning of the inertial device through the SVIVO™ software (Real Track Systems, Almería, Spain). Synchronization of these devices via ANT technology with GARMIN™ heart rate monitors enabled continuous monitoring of heart rate throughout the sessions, providing detailed information on the physiological response to exertion. All collected data were stored locally for subsequent analysis.

The inertial devices used (WIMU Pro™, Real Track Systems, Almería, Spain) have been validated in previous studies demonstrating their reliability and accuracy for quantifying internal, external, and tactical load variables across different sports contexts [32,46,47]. Furthermore, prior to the intervention, their accuracy was verified in the specific context of this study by calculating the error margin of the local positioning system (LPS), which resulted in a maximum value of 3.5 cm, within the acceptable range for research in controlled environments.

All data recordings were processed and converted into quantitative variables using SPRO™ software (Real Track Systems, Almería, Spain), which allows for the processing, export, and interpretation of key metrics such as neuromuscular load (Player Load), distance, speed, number of impacts, and cardiac variables, providing a comprehensive profile of the physical demands experienced by each student.

### 2.5. Procedure

All procedures were conducted in accordance with the Declaration of Helsinki, respecting the voluntary participation and anonymity of the subjects. Initially, approval was obtained from the University’s Bioethics and Biosafety Committee (159/2022). Subsequently, the school administration, teaching staff, and legal guardians of the students were informed about the study’s objectives and characteristics. Prior to the intervention, all necessary permissions and informed consents were secured to carry out the research.

All devices were calibrated and individually configured prior to each assessment session. In addition, the UWB antennas were placed on the playing field for data collection. An initial evaluation (Pre-test) was conducted, consisting of five rounds of three simultaneous matches with two minutes of rest between rounds. Therefore, a total of 15 matches were played. A reduced playing area was used to ensure that all teams could participate simultaneously in three equally sized spaces (20 × 13 m). Teams were composed of four students each (4 vs. 4), and all teams were mixed gender. Each match lasted five minutes. The role of referee was fulfilled by the researchers.

All devices (WIMU Pro™, Real Track Systems, Almería, Spain) were temporally synchronized via satellite signal prior to the start of the session and are equipped with an auto-recording function that is activated when each unit is removed from the charging case for placement on the participant. At the beginning of each specific activity, a timestamp was recorded, which subsequently allowed precise alignment and synchronization of all data obtained from the different sensors and heart rate monitors during data analysis.

**Figure 3:**
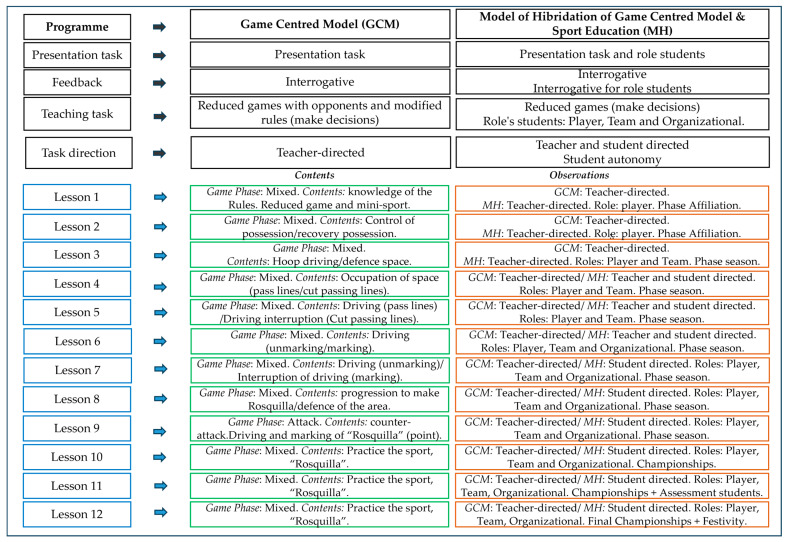
Structure of the GCM and MH intervention programs. Note: Adapted from Calle et al. [13].

Subsequently, 12 sessions of the intervention programs were conducted over a period of two months, each based on a different pedagogical model: the Hybrid Model (HM) and the Game-Centered Model (GCM). All sessions, including the pre-test and post-test, were held on an indoor futsal court within the educational center. The team configurations remained consistent throughout the interventions. Regarding the intervention programs, the GCM group utilized modified and game-centered sport activities designed to promote students’ active participation. The sessions and tasks were teacher-led, incorporating questioning-based feedback provided by the teacher. In the HM program, modified and game-centered sport activities were also implemented with questioning feedback. This approach was combined with the introduction of structured phases: affiliation, pre-season, regular season, final competition, activity record-keeping, and culminating event or festivity. Sessions 1–3 were teacher-led, with students acting as players. In sessions 4–6, leadership was shared between the teacher and the students, who assumed both player and team roles. Sessions 7–12 were fully student-led, with students taking on the roles of players, roles of team, and roles of organizers. The structure of the intervention programs according to the pedagogical models is presented in Figure 3.

After implementing the two intervention programs, a final evaluation (post-test) was conducted with the same characteristics as the pre-test. Five rounds of three simultaneous 4 vs. 4 matches were played, each lasting five minutes, with two minutes of rest between matches. Therefore, a total of 15 matches were played. In the Hybrid Model (HM), the role of referee was performed by the students. The process of recording the variables followed the procedure established by Rico-González et al. [48]. After data collection using inertial devices, data were extracted for subsequent statistical analysis. Following the recommendations of Rico-González et al. [48], data obtained through localization technologies and inertial sensors should be interpreted with caution, considering that the quality of data collection, processing, analysis, and reporting can affect the validity of the results. In this study, the error margin of the local positioning system (LPS) used was previously calculated, yielding a maximum positioning error of 3.5 cm, which falls within acceptable ranges for research in controlled sports environments.

**Figure 4:**
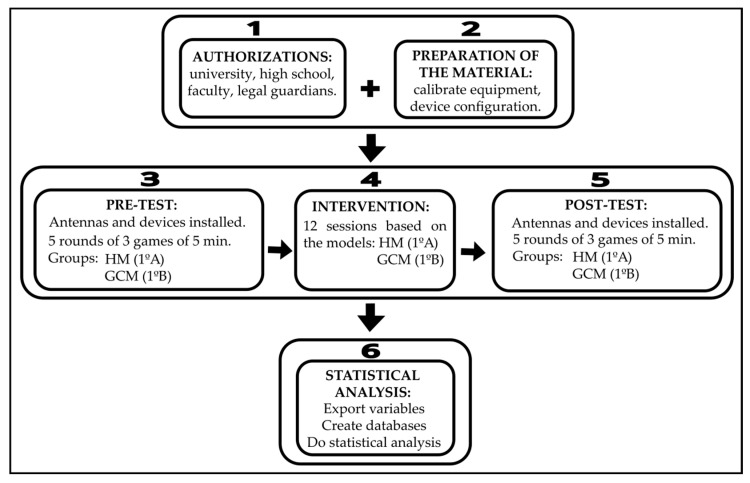
The Procedure developed during the study.

The variable registration system using the ultra-wideband (UWB) system has been validated by Bastida-Castillo et al. [47], who reported that the margin of error is acceptable for research purposes. All participants wore WIMU Pro™ inertial devices (Real Track Systems, Almería, Spain) in combination with GARMIN™ heart rate monitors (Garmin Ltd., Olathe, KS, USA) during the pre-test and post-test assessment sessions. The inertial device was placed on the upper back, specifically in the interscapular line, using a custom-fitted harness to ensure stability during movement. Finally, the recorded variables were converted into quantitative data using SPRO™ software and exported to Jamovi for statistical analysis. The pre-test and post-test followed a similar format, consisting of five rounds of three simultaneous matches, each lasting five minutes. The pre-test was implemented identically across all groups: students assumed the role of players, while teachers and researchers refereed the matches and were responsible for the organization of the sessions. However, in the post-test for the GCM group, the matches were directed and refereed by the teachers and researchers, with students acting solely as players. In contrast, in the post-test for the MH group, students took on the roles of players, roles of team, and roles of organizers. Therefore, the session was organized by the students themselves. Figure 4 presents the phases of the procedure followed in this study.

Table 1 shows differences between the two pedagogical models, HM and GCM, during this study.

### 2.6. Statistical Analysis

Data recorded by the WIMU Pro™ inertial devices and GARMIN™ heart rate monitors were converted into quantitative variables using SPRO™ software. Subsequently, Kolmogorov–Smirnov (normality), Runs test (randomness), and Levene’s test (homogeneity) were performed [49]. Based on the results of these tests, the use of parametric or non-parametric statistical tests was determined depending on the variables. A descriptive analysis was conducted for all variables.

It was verified that controlling for the random effect of individual participant responses was unnecessary, as no improvements in the conditional R^2^ were observed across all variables in the Mixed Linear Model (MLM). Furthermore, no significant effects were found for any variables, indicating that controlling for inter-subject variability was not required [50,51].

Within the inferential analyses, for the comparison of independent paired samples, the t-Student test was applied for parametric variables, and the U Mann–Whitney test for non-parametric variables [49], to compare variables obtained at the initial evaluation between the two models and at the final evaluation between both models. Regarding the comparison of related paired samples, one-way ANOVA with the Welch test was used for parametric variables, and one-way ANOVA with the Kruskal–Walli test for non-parametric variables, to study the evolution of variables within each pedagogical model between the initial evaluation (pre-test) and the final evaluation (post-test) [49]. Differences in means of all variables regarding model (HM and GCM), time point (initial and final evaluation), and the interaction between model and time point were determined through ANOVA and Tukey’s post hoc multiple comparison test [49].

Effect size was calculated using d-Cohen and partial eta squared (*η^2^*) [52]. The effect sizes (d-Cohen) were interpreted as follows: small (0.0–0.2), moderate (0.2–0.6), large (0.6–1.2), and very large (>1.2). Regarding *η^2^* effect size, values were classified as small (0.010–0.059), medium (0.060–0.139), and large (>0.140) [53,54].

All analyses were performed using Jamovi software version 2.3 (The Jamovi Project, Sydney, Australia, 2022).

## 3. Results

**Figure 5:**
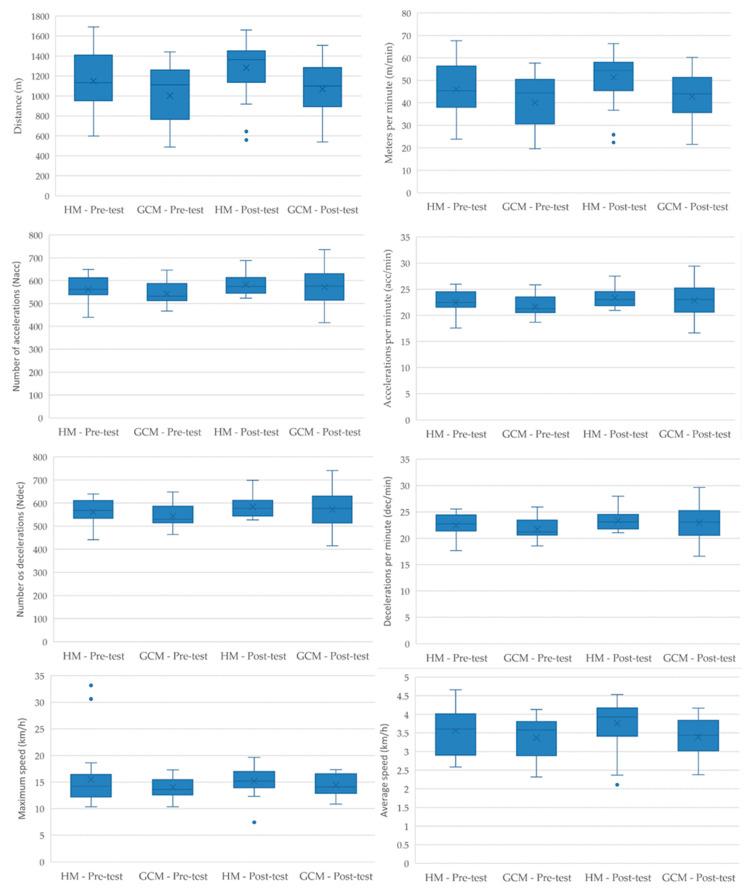

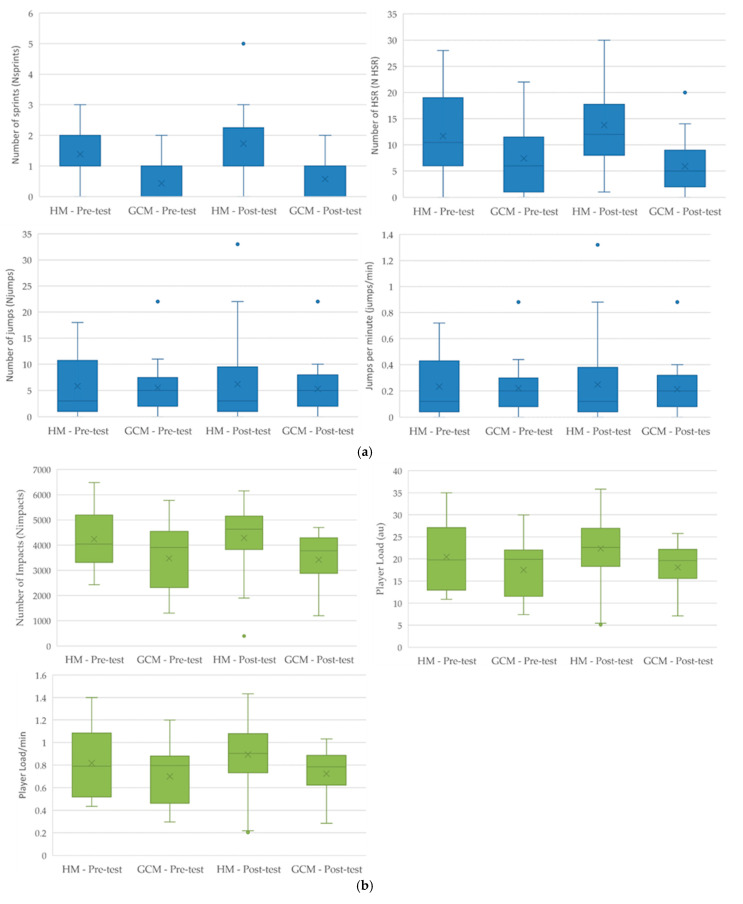

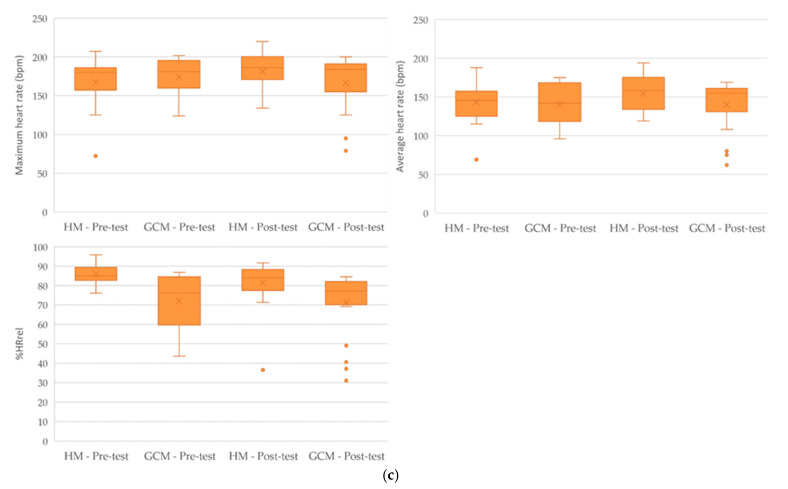
Descriptive Analysis of eTL and iTL Variables According to Pedagogical Models and Evaluation Tests (means and standard deviations for each variable); (**a**) Descriptive Analysis of eTL Kinematic; (**b**) Descriptive Analysis of eTL Neuromuscular; (**c**) Descriptive Analysis of iTL Objective. Note: GCM = Game-Centered Model; MH = Hybrid Model of the Game-Centered Model and Sports Education Model.

The descriptive results of the eTL (kinematic and neuromuscular) and iTL variables analyzed according to each pedagogical model are presented in Figure 5.

Table 2 presents the means and standard deviations of all the analyzed variables, differentiated between the initial and final evaluations according to the pedagogical model.

Differences in all variables between evaluations (initial and final) for each pedagogical model are included in Table 3, which presents the paired sample comparisons, indicating the evolution of variables within each pedagogical model between the initial and final evaluations. Regarding the results, no significant differences were found between the evaluation tests for either pedagogical model.

The differences in eTL and iTL variables with respect to the pedagogical models for each testing moment (pre-test and post-test) are presented in Table 4. In the pre-test, no significant differences were found between the two models in most variables, indicating group homogeneity. However, significant differences were observed in the number of sprints, number of impacts, and percentage of relative heart rate, all of which were higher in the Hybrid Model (HM). In the post-test, the Hybrid Model (HM) showed significantly higher values in multiple variables, both external and internal load, indicating a greater physiological stimulus compared to the Game-Centered Model (GCM).

The results of the ANOVA analysis (Table 5) revealed statistically significant differences for the model factor in several variables, particularly those related to the volume and intensity of physical effort. Significant effects were also found for the time factor in specific kinematic variables, while no significant interactions between model and time were observed.

## 4. Discussion

The aim of the present study was to quantify and compare the physical and physiological demands (eTL and iTL) experienced by students during the practice of an alternative sport, “Rosquilla,” based on the pedagogical model applied (GCM and HM) and the evaluation moment (pre-test/post-test). The descriptive analysis revealed that the Hybrid Model (HM) generated higher values in both kinematic and neuromuscular variables (eTL), as well as in internal load indicators (iTL), compared to the Game-Centered Model (GCM).

In the initial evaluation (pre-test), significant differences were found favoring the Hybrid Model (HM) in the number of impacts and the percentage of relative heart rate, while the remaining variables displayed homogeneous values between groups. It is noteworthy that the groups were naturally occurring and not experimentally manipulated, as they were assigned by the educational administration, which accounts for the minor initial differences. Similar studies did not report significant differences in these variables at the beginning of their interventions [6,18,38].

In the final evaluation (post-test), the Hybrid Model (HM) demonstrated significantly higher values in multiple kinematic variables (including total distance, explosive distance, distance per minute, average speed, number of sprints, HSR, number of steps, and steps per minute), neuromuscular variables (number of impacts, Player Load, and Player Load per minute), and internal load (% relative heart rate), suggesting a higher overall physical stimulus. However, no significant differences were found between pre-test and post-test within each model, indicating that the program duration may not have been sufficient to produce detectable physiological adaptations. These findings are consistent with previous studies in school basketball and football contexts [6,18], which demonstrate that appropriately implemented sports generate relevant physical–physiological demands. Factors such as task design, playing area, type of opposition, and teaching approach clearly influence eTL and iTL levels [55]. Pedagogical and organizational variables that define a task directly affect the eTL during training [56].

Some variables, such as sprints or HSR, showed low values because the thresholds defined in the SPRO™ software (24 km/h for sprint, 21 km/h for HSR) are rarely achieved in school settings. Students find it challenging to attain these speed thresholds during physical education sessions due to their physical characteristics, the dimensions of available playing spaces, and the nature of the sport practiced [57]. In “Rosquilla,” players cannot move while holding the hoop; they can only pass it and create space by moving to occupy different positions.

Previous literature recommends the use of the Game-Centered Model (GCM) over the traditional model due to its benefits for physical fitness [6,38]. However, our results indicate that the Hybrid Model (HM) outperforms the GCM in most kinematic variables (eTL), neuromuscular variables (eTL), and heart rate measures (iTL). Therefore, the hybridization of the GCM with the Sport Education Model (SEM) is more effective in meeting students’ physical and physiological demands. The autonomy granted to students, their assigned roles, and the structured learning situation (phases: affiliation, preseason, season, and festivity), far from reducing intensity, represent an additional motivational factor in the classroom [58,59] that may influence the intensity observed in the final evaluation.

It is important to note that task design, the sport utilized, and the characteristics of the educational context influence practical possibilities, thereby affecting eTL and iTL variables [38]. In our case, the duration of matches comprising each evaluation test was five minutes, which significantly impacts task intensity for schoolchildren. Conversely, matches played on small fields allow multiple games to be conducted simultaneously, enabling all students to participate concurrently. However, this reduces the physical–physiological demands concerning kinematic and neuromuscular variables (eTL). Moreover, the school context imposes practical limitations such as large student numbers, limited space, and restricted class time—factors that constrain task intensity [6,7].

The results show no significant differences in the evaluation tests within each model. Sports experience contributes to improvements in eTL [60], stemming from the physical skills acquired during intervention programs. Evidence demonstrates that athletes’ sports experience influences the number of accelerations performed [61]. The absence of prior experience with the alternative sport practiced indicates that both groups start from a similar level of physical fitness, which explains the similarity in recorded physio-physiological demands. The duration of the intervention programs is insufficient to produce significant improvements. The lack of significant differences between pre-test and post-test may also result from the short program duration (12 sessions). Studies such as Islas-Guerra et al. [62] recommend programs exceeding 12 weeks to enhance physical fitness, given that the tasks in the initial and final evaluations are identical (4 vs. 4 matches) and conducted under equivalent conditions, which does not influence the obtained results.

The Sport Education Model applied throughout a full season produces significant improvements in physical fitness [25]. The design and complexity of tasks, playing area, type of opposition, and type and duration of play are elements that define eTL and iTL [55]. In this study, these elements were identical in the initial and final evaluations, contributing to similar values in eTL and iTL variables and the absence of significant differences. Within the initial evaluation, no significant differences were recorded in most variables when comparing the two pedagogical models, except for the number of impacts and the percentage of relative heart rate.

During the post-test, significant differences appear in kinematic eTL variables (distance, explosive distance, distance per minute, average speed, number of sprints, HSR, number of steps, and steps per minute), neuromuscular eTL variables (number of impacts, Player Load, Player Load per minute), and in the percentage of relative heart rate of iTL, all higher in the Hybrid Model (HM). The differences favoring the HM cannot be attributed to external conditions, as both classes perform identical tasks under the same conditions. Therefore, these differences result from the greater level of engagement and motivation generated by the Hybrid Model [27,63]. The absence of significant differences in maximum and average heart rate is explained by the fact that the tasks during the final evaluation are identical for each group, resulting in similar intensity levels for students in both groups.

Following implementation of the intervention programs, students from both groups gained experience in practicing the alternative sport “Rosquilla.” The type of experience provided by each model influences the nature of student participation and engagement, impacting the development of the final evaluation and generating higher levels of kinematic and neuromuscular eTL for the Hybrid Model (HM). The HM produces superior effects compared to when the two models are used separately, due to increased student participation and enjoyment [63]. Additionally, the differences are influenced by the self-determined motivation generated by the HM through inclusion of the Sport Education Model (SEM). The HM satisfies Basic Psychological Needs [27,64], which in turn leads to higher levels of intrinsic motivation [65], promoting self-determined participation [65,66]. Findings by Wallhead et al. [67,68] demonstrate that improvements in student motivation through SEM application contribute to greater student participation and engagement.

Finally, although both models generated similar levels of average and maximum heart rate, both appear sufficient to improve aerobic fitness, which is relevant given that for many schoolchildren, physical education represents their only source of weekly physical activity [69]. Other studies have shown that active methodologies such as the Game-Centered Model (GCM) involve higher intensity compared to the traditional direct instruction model [6,18,38]. It is necessary to establish high-intensity levels in physical education sessions since many students only engage in physical activity at school. In our case, both models provide equivalent benefits regarding schoolchildren’s aerobic fitness.

## 5. Conclusions

The results of this study indicate that implementing a hybrid instructional approach integrating the Sport Education Model (SEM) and the Game-Centered Model (GCM) elicits greater physical and physiological demands compared to the exclusive application of the GCM. This effect is particularly pronounced in variables related to the kinematic and neuromuscular components of external load.

Although the final assessment tasks are identical across both models, the group exposed to the Hybrid Model (HM) demonstrates significantly higher motor engagement. This increased involvement may be attributed to enhanced motivation and participation fostered by the methodological structure of the HM, which integrates active roles, learner autonomy, and sequential learning phases.

No significant improvements are observed between the pre-test and post-test in either group, suggesting that a 12-session intervention may be insufficient to induce measurable physiological adaptations. Furthermore, the similarity of conditions between the pre-test and post-test limits the sensitivity to detect meaningful changes in the analyzed variables.

Consequently, it is recommended to implement longer-duration pedagogical interventions incorporating active pedagogical models such as the Hybrid Model (HM) to promote sustained improvements in students’ physical fitness within the context of school physical education.

This study demonstrates the added value of using inertial sensors and heart rate monitoring devices in educational settings. Inertial devices enable precise and non-invasive monitoring of the physical loads experienced by students, facilitating pedagogical decision-making based on objective evidence. This technological integration not only enables task adaptation according to actual physiological responses but also expands educators’ assessment capabilities, ultimately optimizing the quality of the teaching–learning process in physical education.

### 5.1. Practical Applications

The results of this study suggest that practicing an alternative invasion sport such as “Rosquilla” can generate physical–physiological demands comparable to those of traditional sports like soccer or basketball in school settings. These findings indicate that “Rosquilla” is a viable, engaging, and inclusive teaching resource within PE programming.

The implementation of the HM, which combines elements of the SEM and the GCM, is associated with higher levels of external load (kinematic and neuromuscular) and internal load, which can be attributed to greater student engagement and motivation due to the active, autonomous, and participatory structure of this methodological approach.

From a practical perspective, these findings support the use of the HM in school settings to optimize PE session intensity, promote the acquisition of motor skills in meaningful contexts, and encourage adherence to regular physical activity.

Furthermore, the need to design longer-duration programs is emphasized to generate sustained improvements in students’ physical fitness, particularly when aiming to impact physiological variables measurable through inertial sensor and heart rate technology.

### 5.2. Study Limitations

This study presents several limitations that should be considered when interpreting the results. First, although the sample size was sufficient for statistical analysis, it may limit the generalizability of the findings to other educational contexts.

Second, the structural characteristics of the school environment (available space, limited class duration, and academic calendar) constrain the actual intensity of tasks and hinder the sustained implementation of innovative pedagogical programs. These characteristics cannot be controlled due to the particularities of the Spanish educational system, where a limited number of weekly class hours are assigned to the PE area, although teachers can determine the number of sessions for each learning situation based on knowledge domains and competencies to be developed. Long-term interventions are required to achieve meaningful learning related to physical fitness. Moreover, the groups were not configured with an equal number of participants, since within the Spanish educational system, the educational administration organizes groups based on educational and equity criteria. It was deemed necessary to respect the configuration established by the administration to ensure an ecological context. Therefore, the natural and unequal structure of the groups may influence the research outcomes. Consequently, future research should consider experimental designs with balanced groups in terms of the number of participants.

Another relevant limitation is that the physical–physiological load was quantified exclusively during evaluation tests (pre-test and post-test) due to time constraints that prevented continuous sensor use throughout all intervention sessions. This limits precise understanding of the intra-program evolution of experienced load.

Among future research directions, it is considered necessary to compare the physical–physiological demands during intervention program tasks. However, the need to maximize the limited time available for each intervention session restricted the use of measurement devices to evaluation tests only. Additionally, it would be advisable to assess the physical–physiological demands throughout multiple interventions to verify improvements in long-term programs while expanding the sample size.

## Figures and Tables

**Table 1:** Comparative table of the pedagogical models during the study.

			HM	GCM
Participants			26 student (class A) (10 boys and 16 girls)	21 students (class B) (11 boys y 10 girls)
Teaching function	Pre-test		Teacher-directed+ arbitrated	Teacher-directed+ arbitrated
	IP	S1–S3	Teacher-directed	Teacher-directed
		S4–S6	Teacher and student-directed	Teacher-directed
		S7–S12	Guide	Teacher-directed
	Post-test		Guide	Teacher-directed+ arbitrated
Student function	Pre-test		Player role	Player role
	IP	S1–S3	Player role	Player role
		S4–S6	Player and team role	Player role
		S7–S12	Player, team, and organizational roles	Player role
	Post-test		player, team, and organizational roles	Player role
Procedure	Pre-test		5 rounds: 3 matches (4 vs. 4) of 5 min.	5 rounds: 3 matches (4 vs. 4) of 5 min.
	IP	S1	Affiliation phase + rules	Rules and team formation
		S2–S9	Preseason phase, modified games	Modified games with opponent
		S10–S11	Regular champion phase (mini sport)	Regular champion (mini sport)
		S12	Final champion phase and festivity	Final round
	Post-test		5 rounds: 3 matches (4 vs. 4) of 5 min.	5 rounds: 3 matches (4 vs. 4) of 5 min.

Note: IP = intervention programs; S = session.

**Table 2:** Descriptive Analysis of Variables in the Initial and Final Evaluations According to the Pedagogical Model.

Variables		Pre-Test		Post-Test	
		HM M ± SD	GCM M ± SD	HM M ± SD	GCM M ± SD
Kinematic eTL	Distance (m)	1150 ± 302	1002 ± 305	1283 ± 275	1069 ± 281
	Explosive Distance (m)	116 ± 62.3	99.6 ± 58.4	125 ± 52.1	92.1 ± 51.2
	Distance/Minute (m/min)	46 ± 12.1	40.1 ± 12.2	51.3 ± 11.0	42.8 ± 11.3
	Number of Accelerations (Nacc)	561 ± 55.3	542 ± 47.2	584 ± 45.1	572 ± 78.4
	Accelerations/Minute (acc/min)	22.4 ± 2.21	21.7 ± 1.89	23.3 ± 1.80	22.9 ± 3.14
	Number of Decelerations (Ndec)	561 ± 54	543 ± 47.9	584 ± 44.9	573 ± 78.1
	Decelerations/Minute (dec/min)	22.5 ± 2.16	21.7 ± 1.92	23.3 ± 1.80	22.9 ± 3.12
	Max. Speed (km/h)	15.5 ± 5.38	14 ± 2.05	15.1 ± 2.46	14.5 ± 2.06
	Average Speed (km/h)	3.56 ± 0.60	3.37 ± 0.57	3.76 ± 0.60	3.39 ± 0.50
	Number of Sprints (Nsprints)	1.38 ± 0.94	0.43 ± 0.68	1.73 ± 1.08	0.57 ± 0.81
	HSR (High-Speed Running) (N HSR)	11.7 ± 8.02	7.43 ± 7.23	13.8 ± 6.99	5.90 ± 5.44
	Number of Steps (Nsteps)	1246 ± 519	1006 ± 483	1377 ± 536	1033 ± 420
	Steps/Minute (steps/min)	49.9 ± 20.8	40.3 ± 19.3	55.1 ± 21.4	41.3 ± 16.8
	Number of Jumps (Njumps)	5.85 ± 5.74	5.52 ± 5.00	6.23 ± 7.82	5.33 ± 4.78
	Jumps/Minute (jumps/min)	0.23 ± 0.23	0.22 ± 0.20	0.25 ± 0.31	0.21 ± 0.19
Neuromuscular eTL	Number of Impacts (Nimpacts)	4239 ± 1206	3475 ± 1344	4286 ± 1291	3418 ± 1109
	Player Load (au)	20.5 ± 7.16	17.5 ± 6.69	22.4 ± 7.83	18.1 ± 5.57
	Player Load/Minute (PL/min)	0.82 ± 0.29	0.70 ± 0.27	0.89 ± 0.31	0.72 ± 0.22
Internal load iTL	Maximum Heart Rate (bpm)	168 ± 34.3	174 ± 24	181 ± 28.5	167 ± 39.7
	Average Heart Rate (bpm)	143 ± 29.1	141 ± 26.6	154 ± 27.7	140 ± 32.1
	% Relative Heart Rate (%)	86 ± 5.26	72.1 ± 13.2	81.6 ± 10.9	71.2 ± 16.6

Note: HM = Hybrid Model of GCM and SEM; GCM = Game-Centered Model; *M* = mean; *SD* = standard deviation.

**Table 3:** Differences in variables between evaluation tests (Pre-test—Post-test) according to Pedagogical Model.

Variables		HM/ *F/X*^2^	*p*	*η* ^2^	GCM *F/X*^2^	*p*	*η* ^2^
Kinematic eTL	Distance (m)	2.74	0.10	0.05	0.54	0.47	0.01
	Explosive Distance (m)	0.32	0.57	0.01	0.19	0.66	0.004
	Distance/Minute (m/min)	2.74	0.10	0.05	0.54	0.47	0.01
	Number of Accelerations (Nacc)	2.57	0.11	0.05	2.21	0.15	0.06
	Accelerations/Minute (acc/min)	2.57	0.11	0.05	2.21	0.15	0.06
	Number of Decelerations (Ndec)	2.60	0.11	0.05	2.34	0.14	0.07
	Decelerations/Minute (dec/min)	2.60	0.11	0.05	2.34	0.14	0.07
	Max. Speed (Km/h)	0.08	0.77	0.002	0.44	0.51	0.01
	Average Speed (Km/h)	1.41	0.24	0.03	0.01	0.90	<0.001
	Number of Sprints (Nsprints)	1.52	0.22	0.03	0.24	0.62	0.01
	HSR (High-Speed Running) (N HSR)	0.99	0.32	0.02	0.59	0.44	0.02
	Number of Steps (Nsteps)	0.79	0.38	0.02	0.03	0.85	<0.001
	Steps/Minute (steps/min)	0.79	0.38	0.02	0.03	0.85	<0.001
	Number of Jumps * (Njumps)	0.06	0.81	<0.001	0.02	0.90	<0.001
	Jumps/Minute * (jumps/min)	0.06	0.81	<0.001	0.02	0.90	<0.001
Neuromuscular eTL	Number of Impacts (Nimpacts)	0.02	0.89	<0.001	0.02	0.88	<0.001
	Player Load (au)	0.85	0.36	0.02	0.09	0.76	0.002
	Player Load/Minute (PL/min)	0.85	0.36	0.02	0.09	0.76	0.002
Internal load iTL	Maximum Heart Rate (bpm)	2.41	0.13	0.05	0.57	0.45	0.02
	Average Heart Rate (bpm)	1.95	0.17	0.04	0.01	0.94	<0.001
	% Relative Heart Rate * (%)	3.46	0.07	0.09	0.03	0.85	<0.001

Note: HM = Hybrid Model of GCM and SEM; GCM = Game-Centered Model; *F* = one-way ANOVA statistic for parametric samples (Welch); *χ*^2^ = one-way ANOVA statistic for non-parametric samples (Kruskal–Wallis); * non-parametric variables.

**Table 4:** Differences in Variable Outcomes Among Pedagogical Models According to the Evaluation Test.

Variables		Pre-Test *t/U*	*p*	*d* *Cohen*	Post-Test *t/U*	*p*	*d* *Cohen*
Kinematic eTL	Distance (m)	−1.66	0.10	0.49	−2.63	0.01 **	0.6
	Explosive Distance (m)	−0.90	0.37	0.27	−2.14	0.03 **	0.63
	Distance/Minute (m/min)	−1.66	0.10	0.48	−2.63	0.01 **	0.76
	Number of Accelerations (Nacc)	−1.27	0.21	0.37	0.66	0.51	0.19
	Accelerations/Minute (acc/min)	−1.27	0.21	0.34	0.66	0.51	0.16
	Number of Decelerations (Ndec)	−1.24	0.22	0.36	−0.57	0.57	0.17
	Decelerations/Minute (dec/min)	−1.24	0.22	0.35	−0.57	0.57	0.16
	Max. Speed (Km/h)	−1.17	0.25	0.37	−1.01	0.31	0.26
	Average Speed (Km/h)	−1.11	0.27	0.32	−2.27	0.03 **	0.66
	Number of Sprints * (Nsprints)	120	<0.001 **	1.15	105	<0.001 **	1.2
	HSR (High-Speed Running) (N HSR)	−1.89	0.06	0.56	−4.22	<0.001 **	1.2
	Number of Steps (Nsteps)	−1.62	0.11	0.48	−2.40	0.02 **	0.71
	Steps/Minute (steps/min)	−1.62	0.11	0.48	−2.40	0.02 **	0.72
	Number of Jumps (Njumps)	−0.20	0.84	0.06	−0.46	0.64	0.14
	Jumps/Minute (jumps/min)	−0.20	0.84	0.05	−0.46	0.64	0.15
Neuromuscular eTL	Number of Impacts (Nimpacts)	−2.05	0.04 **	0.59	−2.44	0.02 **	0.72
	Player Load (au)	−1.44	0.16	0.43	−2.10	0.04 **	0.63
	Player Load/Minute (PL/min)	−1.44	0.16	0.42	−2.10	0.04 **	0.63
Internal load iTL	Maximum Heart Rate * (bpm)	0.76	0.45	0.2	215	0.21	0.40
	Average Heart Rate (bpm)	−0.31	0.76	0.07	−1.63	0.11	0.47
	% Relative Heart Rate * (%)	−4.94	<0.001 **	1.38	131	0.002 **	0.74

Note: MH = Hybrid Model of GCM and SEM; GCM = Game-Centered Model; *t* = t-Student; *U* = U Mann–Whitney; * = non-parametric variables; ** = *p* < 0.05.

**Table 5:** Differences in variables with respect to the pedagogical model, evaluation test, and the interaction between the model and evaluation test.

Variables		Factors	*F*	*p*	*η* *^2^*	Post Hoc
Kinematic eTL	Distance (m)	Model	9.00	0.003 *	0.09	a
		Moment	2.73	0.10	0.03	-
		Model × Moment	0.30	0.58	0.01	f
	Explosive Distance (m)	Model	4.32	0.04 *	0.04	a
		Moment	0.01	0.947	0.00	-
		Model × Moment	0.49	0.48	0.005	
	Distance/Minute (m/min)	Model	9.00	0.003 *	0.09	a
		Moment	2.73	0.10	0.03	-
		Model × Moment	0.30	0.58	0.01	f
	Number of Accelerations (Nacc)	Model	1.76	0.19	0.02	-
		Moment	4.82	0.03 *	0.05	b
		Model × Moment	0.09	0.76	0.01	-
	Accelerations/Minute (acc/min)	Model	1.76	0.19	0.02	-
		Moment	4.82	0.03 *	0.05	b
		Model × Moment	0.09	0.76	0.01	-
	Number of Decelerations (Ndec)	Model	1.49	0.22	0.02	-
		Moment	5.01	0.03 *	0.052	b
		Model × Moment	0.125	0.72	0.01	-
	Decelerations/Minute (dec/min)	Model	1.49	0.22	0.02	-
		Moment	5.01	0.03 *	0.052	b
		Model × Moment	0.12	0.72	0.01	-
	Max. Speed (Km/h)	Model	2.27	0.13	0.025	-
		Moment	0.01	0.95	0.00	-
		Model × Moment	0.29	0.59	0.003	-
	Average Speed (Km/h)	Model	5.60	0.02 *	0.06	a
		Moment	0.83	0.36	0.01	-
		Model × Moment	0.56	0.45	0.01	-
	Number of Sprints (Nsprints)	Model	31.80	<0.001 *	0.26	a
		Moment	1.70	0.20	0.01	-
		Model × Moment	0.29	0.60	0.01	d, e, f, g.
	HSR (High-Speed Running) (N HSR)	Model	17.21	<0.001 *	0.16	a
		Moment	0.03	0.85	0.00	-
		Model × Moment	1.52	0.221	0.01	d, e, f.
	Number of Steps (Nsteps)	Model	8.06	0.006 *	0.08	a
		Moment	0.58	0.45	0.01	-
		Model × Moment	0.26	0.61	0.003	-
	Steps/Minute (steps/min)	Model	8.06	0.006 *	0.08	a
		Moment	0.58	0.45	0.01	-
		Model × Moment	0.26	0.61	0.003	-
	Number of Jumps (Njumps)	Model	0.23	0.63	0.003	-
		Moment	0.01	0.94	0.00	-
		Model × Moment	0.05	0.82	0.001	-
	Jumps/Minute (jumps/min)	Model	0.23	0.63	0.003	-
		Moment	0.01	0.94	0.00	-
		Model × Moment	0.05	0.82	0.001	-
Neuromuscular eTL	Number of Impacts (Nimpacts)	Model	10.02	0.002 *	0.100	a
		Moment	<0.001	0.98	0.00	-
		Model × Moment	0.04	0.84	0.00	-
	Player Load (au)	Model	6.26	0.01 *	0.06	a
		Moment	0.75	0.39	0.01	-
		Model × Moment	0.22	0.64	0.002	-
	Player Load/Minute (PL/min)	Model	6.26	0.01 *	0.06	a
		Moment	0.75	0.39	0.01	-
		Model × Moment	0.22	0.64	0.002	-
Internal load iTL	Maximum Heart Rate * (bpm)	Model	0.33	0.56	0.004	-
		Moment	0.20	0.66	0.002	-
		Model × Moment	2.55	0.11	0.03	-
	Average Heart Rate (bpm)	Model	1.95	0.17	0.02	-
		Moment	0.75	0.39	0.01	-
		Model × Moment	0.94	0.33	0.01	-
	% Relative Heart Rate (%)	Model	24.38	<0.001 *	0.21	a
		Moment	1.15	0.29	0.01	-
		Model × Moment	0.52	0.47	0.01	d, e, f, g.

Note: HM = Hybrid Model combining GCM and SEM; GCM = Game-Centered Model; *F* = ANOVA statistic; *η*^2^ = Eta-squared; a = GCM–HM; b = post–pre; d = post GCM–post HM; e = post GCM–pre HM; f = pre GCM–post HM; g = pre GCM–pre HM; * *p* < 0.05.

## Data Availability

Data are contained within the article.
